# Mendelian Randomisation study of the influence of eGFR on coronary heart disease

**DOI:** 10.1038/srep28514

**Published:** 2016-06-24

**Authors:** Pimphen Charoen, Dorothea Nitsch, Jorgen Engmann, Tina Shah, Jonathan White, Delilah Zabaneh, Barbara Jefferis, Goya Wannamethee, Peter Whincup, Amy Mulick Cassidy, Tom Gaunt, Ian Day, Stela McLachlan, Jacqueline Price, Meena Kumari, Mika Kivimaki, Eric Brunner, Claudia Langenberg, Yoav Ben-Shlomo, Aroon Hingorani, John Whittaker, Juan Pablo Casas, Frank Dudbridge, the UCLEB Consortium, Caroline Dale, Chris Finan, Andrew Wong, Ken Ong, Fotios Drenos, Jackie Cooper, Reecha Sofat, Floriaan Schmidt, Debbie A. Lawlor, Philippa J. Talmud, Steve E. Humphries, Rebecca Hardy, Diana Kuh, Nicholas Wareham, Richard Morris, Vincent Plagno

**Affiliations:** 1 Department of Non-communicable Disease Epidemiology, London School of Hygiene and Tropical Medicine, UK; 2 Department of Tropical Hygiene, Faculty of Tropical Medicine, Mahidol University, Thailand; 3 Institute of Cardiovascular Science, University College London, UK; 4 University College London Genetics Institute, Department of Genetics, Environment and Evolution, University College London, UK; 5 Institute of Psychiatry, Psychology & Neuroscience, King’s College London, UK; 6 Department of Primary Care & Population Health, University College London, UK; 7 Population Health Research Institute, St George’s, University of London, UK; 8 MRC Integrative Epidemiology Unit, School of Social and Community Medicine, University of Bristol, UK; 9 Centre for Population Health Sciences, The Usher Institute of Population Health Sciences and Informatics, The University of Edinburgh, UK; 10 Institute for Social and Economic Research, University of Essex, UK; 11 Department of Epidemiology and Public Health, University College London, UK; 12 Farr Institute of Health Informatics, University College London, UK; 13 MRC Epidemiology Unit, School of Clinical Medicine, University of Cambridge, UK; 14 School of Social and Community Medicine, University of Bristol, UK; 15 Genetics Division, Research and Development, GlaxoSmithKline, UK; 16 MRC Unit for Lifelong Health and Ageing, London, UK; 17 MRC Epidemiology Unit, Institute of Metabolic Science, Addenbrooke’s Hospital, Cambridge, UK; 18 Centre for Cardiovascular Genetics, Dept. of Medicine, British Heart Foundation Laboratories, Rayne Building, Royal Free and University College Medical School, London, UK; 19 Centre for Clinical Pharmacology, University College London, London, UK

## Abstract

Impaired kidney function, as measured by reduced estimated glomerular filtration rate (eGFR), has been associated with increased risk of coronary heart disease (CHD) in observational studies, but it is unclear whether this association is causal or the result of confounding or reverse causation. In this study we applied Mendelian randomisation analysis using 17 genetic variants previously associated with eGFR to investigate the causal role of kidney function on CHD. We used 13,145 participants from the UCL-LSHTM-Edinburgh-Bristol (UCLEB) Consortium and 194,427 participants from the Coronary ARtery DIsease Genome-wide Replication and Meta-analysis plus Coronary Artery Disease (CARDIoGRAMplusC4D) consortium. We observed significant association of an unweighted gene score with CHD risk (odds ratio = 0.983 per additional eGFR-increasing allele, 95% CI = 0.970–0.996, p = 0.008). However, using weights calculated from UCLEB, the gene score was not associated with disease risk (p = 0.11). These conflicting results could be explained by a single SNP, rs653178, which was not associated with eGFR in the UCLEB sample, but has known pleiotropic effects that prevent us from drawing a causal conclusion. The observational association between low eGFR and increased CHD risk was not explained by potential confounders, and there was no evidence of reverse causation, therefore leaving the remaining unexplained association as an open question.

Chronic kidney disease (CKD) occurs in 15–20% of the general population aged 65 years or older. Markers of CKD, such as a low estimated glomerular filtration rate (eGFR) and/or elevated Urine Albumin Creatinine Ratio (UACR), are major independent risk factors for cardiovascular and all-cause mortality^1^,^2^. In particular, people with lower eGFR are at a higher risk of developing coronary heart disease (CHD).

Although it has been proposed that CKD may be causally associated with CHD, the precise biological pathways for this association are not well understood, and hypotheses regarding this finding are varied, broadly involving inflammatory pathways and/or vascular calcification^3^,^4^,^5^,^6^. Currently, people with CKD are treated with blood pressure lowering drugs and statins to prevent poor outcomes. However, many genetic variants that are associated with high blood pressure are not associated with eGFR or CKD^7^, as might be expected if they were on the same causal pathway to CHD. Also, there is little overlap in association between CKD and CHD genetic variants and early markers of cardiovascular disease, which would be expected if kidney function were causally related to CHD^8^.

People with CKD tend to have had adverse early life circumstances that predispose them to cardiovascular disease^9^,^10^,^11^, and individuals who are overweight at earlier age are more likely to have CKD at older age suggesting that CKD is a marker or consequence of a cumulative adverse life style. Hence, it is not yet clear whether low kidney function directly causes CHD or whether the observed association is due to other shared risk factors/confounders, in particular socioeconomic status, early life risk factors and unhealthy lifestyle.

Here we applied Mendelian Randomisation (MR) to determine whether lower eGFR per se has a causal role to contribute to later CHD. To our knowledge, this is the first MR study to explicitly investigate the influence of eGFR on CHD. Previously, Olden *et al*. studied whether several genetic variants affect both eGFR and CHD^8^. They identified one SNP, rs653178 that was associated with both; but while this is consistent with a causal effect, they did not consider whether it met the assumptions required for MR nor did they estimate a causal effect size. Here we go further by combining 17 SNPs into a single gene score, which can improve the power of MR studies when individual SNPs are not significantly associated with the outcome^12^, and we use a richly phenotyped data set, the UCLEB consortium^13^ to thoroughly examine the possibility of violating the MR assumptions through confounding or pleiotropy.

## Research design and methods

### Study design

We used data from two consortia, UCLEB^13^ and CARDIoGRAMplusC4D^14^,^15^,^16^. The former allowed assessment of the MR assumptions for eGFR, namely that the gene score is associated with eGFR, not associated with potential confounders of the eGFR-CHD association, and not associated with other biomarkers of CHD that might represent alternative pathways than that through eGFR. In UCLEB, we used individual patient data from 2,249 cases of CHD and 10,896 controls from 7 cohort studies: the British Regional Heart Study (BRHS), British Women’s Heart and Health Study (BWHHS), Caerphilly Prospective Study (CAPS), Edinburgh Type-2 Diabetes Study (ET2DS), Edinburgh Artery Study (EAS), English Longitudinal Study of Ageing (ELSA), and Whitehall II Study (WHII). Four studies (BRHS, BWHHS, CAPS, and ET2DS) have eGFR available. UCLEB has 116 phenotypes related to cardiovascular function, allowing SNPs to be thoroughly assessed for validity of the MR assumptions.

The CARDIoGRAMplusC4D consortium provides a large sample for assessing genetic association with CHD. The Consortium released summary statistics from 3 meta-analyses of coronary artery disease: CARDIoGRAM genome-wide association study (GWAS) with 22,233 CHD cases and 64,762 controls^16^, C4D GWAS with 15,420 CHD cases and 15,062 controls^15^, and the combined data of these two GWAS with additional cohorts, CARDIoGRAMplusC4D Metabochip, with 63,746 CHD cases and 130,681 controls^14^. These consortia data allow the assessment of genetic association with CHD in very large samples, but do not provide information on eGFR.

### Selection of SNPs and construction of gene score

We identified 32 SNPs previously reported to be associated with eGFR (S1 Table). Seventeen of them, which are not in linkage disequilibrium (LD) and have been genotyped in UCLEB, were shortlisted as potential components of an instrument for MR (S2 Table) because they could be investigated for validity of the MR assumptions using the UCLEB data. Due to the different genotyping platforms used in CARDIoGRAM GWAS, C4D GWAS, and CARDIoGRAMplusC4D Metabochip, our 17 selected SNPs are not always available in all 3 meta-analyses. Thus the total sample sizes vary across SNPs used to investigate association with CHD (S1 Fig).

These selected SNPs were then combined into gene scores. Two types of gene score were used: 1) unweighted gene score, defined for subject *i* as 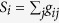 where *g*_*ij*_ is the count of eGFR-increasing alleles for subject *i* at SNP *j* with the direction of effect taken from the UCLEB data, and 2) weighted gene score, 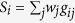, which includes estimates of effects on eGFR, *w*_*j*_, at each SNP based on the UCLEB data. More precisely, the *w*_*j*_ are the estimates from linear regression analyses of eGFR on SNP *j*, under additive genotypic coding.

### Exposure and outcome variables

Our primary outcome is CHD (defined as fatal or non fatal myocardial infarction, or revascularization, from both diagnosis validation and self-report) and the exposure is the level of kidney function measured by eGFR. In the UCLEB data, we derived eGFR based on creatinine using the modification of diet in renal disease (MDRD) model^17^


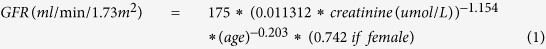


eGFR was normally distributed in our data, therefore no further transformation was applied.

### Potential confounding factors

From the total of 116 traits available in UCLEB, we systematically checked for potential confounding factors across 94 traits measured in up to 7 studies. We identified potential confounders by testing each of these traits for both eGFR-trait (available in 4 studies) and CHD-trait associations. As the presence of both associations is necessary but not sufficient to establish confounding, we considered those associated traits as potential confounding factors, unless there is additional external evidence that a particular biomarker is renally cleared, i.e. potentially on the causal pathway between kidney function and CHD. For the eGFR-trait and CHD-trait associations we controlled the false discovery rate at 0.05 by the Benjamini-Hochberg procedure. We used a permutation procedure to check whether correlation among traits would cause this procedure to be conservative, finding that the overall level of correlation is negligible in this context (see Supplementary Methods).

### SNP instrument validity

We used UCLEB data to test the validity of our gene scores as instrumental variables under the three MR assumptions: 1) association between gene scores and eGFR, 2) absence of association between gene scores and common causes of eGFR and CHD, and 3) absence of pathways between gene scores and CHD other than through eGFR. We tested assumption 1 using linear regression of eGFR on the gene scores, assumption 2 using linear regression of each identified potential confounder on the gene scores (except gender where logistic regression was applied), and assumption 3 using linear regression of each trait in UCLEB (whether or not a potential confounder) on the gene scores. Each regression adjusted for study as a categorical covariate. We controlled the false discovery rate at 0.05 by the Benjamini-Hochberg procedure.

### Mendelian Randomisation

We tested for a causal effect by testing the association between each gene score and CHD and appealing to the MR principle. As only summary odds ratios and their standard errors were available from the CHD consortia, we applied the Johnson formula described by Burgess *et al*.^12^, in which the regression coefficient of the gene score on CHD is


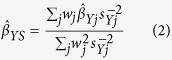


with standard error 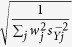, where 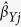 is the estimated log odds ratio of SNP *j* on CHD, *s*_*Yj*_ is its standard error, and *w*_*j*_ is a predetermined weight. The unweighted score has *w*_*j*_ = 1 for all SNPs, whereas the weighted score sets *w*_*j*_ to the linear regression coefficient of SNP *j* for eGFR, here estimated from the UCLEB data (weights and odds ratios were calculated for the eGFR increasing alleles). This is an example of two-sample Mendelian randomization^18^ in which the exposure and outcome associations are measured in different samples. Two-sided tests of association were performed by comparing the ratio of 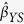 and its standard error to a standard normal distribution.

Using the weighted score, 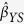 is an estimate of the causal effect of eGFR on CHD, but we avoid this interpretation as our aim here was merely to detect the presence of a causal effect.

### Reverse Mendelian Randomisation

We considered whether a causal effect exists in the opposite direction, with CHD acting to cause reduced kidney function. We constructed an weighted gene score from 51 SNPs associated with CHD at genome-wide significance, as reported by CARDIoGRAMplusC4D^14^ and tested this score for association with eGFR in the UCLEB data using summary odds ratios as above.

## Results

**Figure 1:**
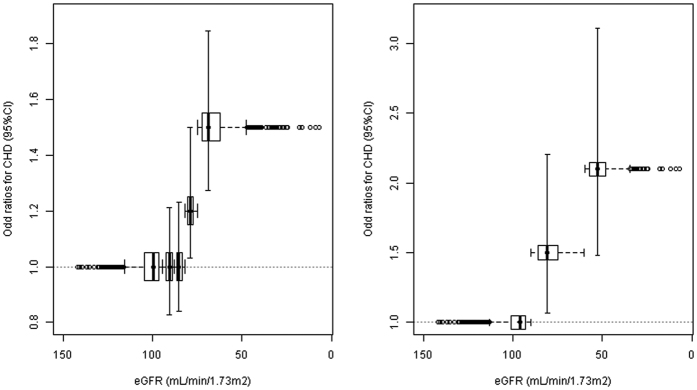
Association between baseline eGFR and CHD in quintiles (left), and comparing low and moderate eGFR to normal/high eGFR (right), in the UCLEB data (1515 cases, 5247 controls).

A summary of CHD cases and controls in UCLEB is shown in Table 1. As expected, we observed that eGFR is significantly negatively associated with CHD (OR = 0.984 per ml/min/1.73 m^2^ increase, 95% CI = 0.979–0.988, p = 1.98 × 10^−12^) across its entire range and particularly when comparing subjects with reduced (<60 ml/min/1.73 m^2^) eGFR to those in the intermediate (60–90 ml/min/1.73 m^2^), and normal/high range (≥90 ml/min/1.73 m^2^, Fig. 1), consistent with previous studies of the MDRD eGFR-CHD association^19^.

**Figure 2:**
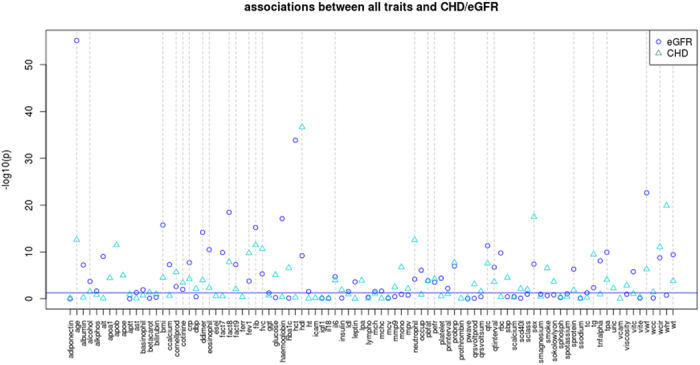
Benjamini-Hochberg adjusted *P*-values for CHD-trait and eGFR-trait (N range of CHD-trait = 139-13145, and N range of eGFR-trait = 138-6764). Horizontal line shows adjusted *P* = 0.05.28 traits significantly associated with both CHD and eGFR on dotted vertical lines are identified as potential confounding factors: age, alcohol, body mass index, ECG Cornell product, cotinine, CRP, D-Dimer, Eosinophils, factor VIII, Factor IX, FEV1, fibrinogen, FVC, HDL, IL-6, Neutrophil, percentage body fat, peak expiratory flow rate, NT-proBNP, ECG QTc, ECG QT interval, sex, total serum protein concentration, Triglyceride, tPa, von Willebrand factor, waist circumference, weight. Abbreviations used in the figure are defined in S3 Table.

From 94 traits available in 7 UCLEB studies, we identified 28 potential confounding factors of the eGFR-CHD association (Fig. 2, S4 Table). These are generally concordant with previously observed correlates of eGFR and risk factors for CHD^19^. Some discrepancies, for example blood pressure and smoking (ever/never), which were not associated with eGFR in UCLEB are likely due to the cross-sectional nature of the data, and have been previously seen in other data^20^. One of the potentially confounding factors was NTproBNP, which is known to be renally cleared^6^. NTproBNP was therefore considered to be a variable on the causal pathway between eGFR and CHD and not a confounding variable. We also excluded percentage body fat because this was only available in the ET2DS study, which did not have many of the other confounders measured. Due to missing data in the remaining 26 potential confounders, we then investigated the observational association between eGFR and CHD in a reduced sample size of 1547 (OR = 0.977 per 1 ml/min/1.73 m^2^ increase, 95% CI = 0.965–0.989, p = 3.38 × 10^−4^). After adjusting for 26 potential confounders, the association between eGFR-CHD was slightly attenuated and remained borderline significant (OR = 0.868 per 10 ml/min/1.73 m^2^ increase, 95% CI = 0.760–1.000, p = 0.05).

**Figure 3:**
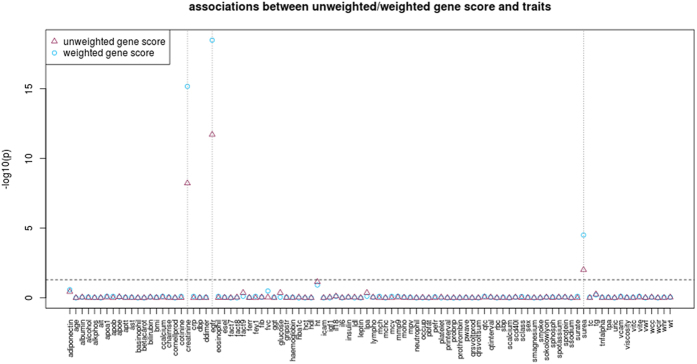
Benjamini-Hochberg adjusted *P*-values for unweighted and weighted gene score-trait associations (N range = 139-15609). Only kidney function traits (eGFR, creatinine, and serum urea concentration) are shown to be significantly associated with either gene score (dotted vertical lines). See S5 Table for further details of summary statistics.

In UCLEB, the combination of 17 SNPs explained 1.5% of the variation in eGFR. The corresponding F statistics were 59 and 91 for unweighted and weighted scores respectively, indicating that the gene score is a strong instrument. In testing for association between gene scores and all traits available in UCLEB (Fig. 3), our gene scores showed an exclusive association with kidney function measurements, including eGFR (adjusted p = 2.0 × 10^−12^ and p = 3.3 × 10^−19^ for unweighted and weighted gene scores respectively), creatinine (adjusted p = 6.0 × 10^−9^ and p = 6.8 × 10^−16^), and serum urea concentration (adjusted p = 9.8 × 10^−3^ and p = 3.1 × 10^−5^). This confirms the first MR assumption that our gene scores are indeed a good proxy for kidney function. Secondly, associations between our gene scores and 26 potential confounding factors and NTproBNP were not significant, meeting the second MR assumption that our gene scores are not associated with common causes of eGFR and CHD. Although we cannot be sure that our gene scores are not associated with unmeasured confounders, these results (including many of the strongest biomarkers for CHD) suggest that any such confounding is weak. Lastly, association between our gene scores and other traits available in UCLEB do not reach significance. Therefore our gene scores appear specific to eGFR and other kidney-related traits.

We found significant association of the unweighted score with CHD (OR = 0.983 per additional eGFR increasing allele, 95% CI = 0.970–0.996, p = 0.008), suggesting a causal effect of eGFR on CHD. This was in the direction to that seen in the observational data. However, the association was not significant when using a weighted score (OR = 0.993, 95% CI = 0.984–1.002, p = 0.11). Inspection of the weights given to the individual SNPs suggested that these results could be explained by rs653178 alone. Previously, it has been shown that there is little overlap between well-validated SNPs for kidney disease and CHD, where only rs653178 was highly associated with both cystatin-C-based eGFR and CHD^21^. However this was not the case in UCLEB, in which rs653178 was not associated with eGFR and received the smallest weight among all 17 instrument SNPs (S6 Table). Therefore, using weights from UCLEB diminished evidence of a causal association observed with the unweighted score.

The unweighted gene score was further investigated to assess the sensitivity of the observed nominal evidence of a causal association. We first excluded rs1260326, which has known pleiotropic effects^22^, and observed the same association signal remaining (OR = 0.991, 95% CI = 0.981–1.001, p = 0.003). Secondly we excluded rs653178 which could have stronger effect through cystatin-based eGFR instead of creatinine-based eGFR^22^ from the gene score, and found that the evidence of causal effect disappeared (OR = 0.994, 95% CI = 0.982–1.005, p = 0.291). This implies that rs653178 is driving the significant association of the gene score with CHD. To confirm this, we excluded each of the 17 SNPs at a time, and observed that only the exclusion of rs653178 removed the association signal. In addition, instead of identifying the eGFR increasing alleles from the UCLEB data, we incorporated external information on the direction of effect for 9 SNPs that were available in Olden *et al*., to allow more accurate estimates due to their larger sample size. While the eGFR increasing alleles in UCLEB are retained for the remaining SNPs, for 3 out of the 9 SNPs from Olden *et al*. a different eGFR increasing allele was identified in comparison to UCLEB (S6 Table). After adjusting the unweighted score accordingly, the evidence of a causal effect remained (OR = 0.971, 95% CI = 0.950–0.992, p = 0.007).

We further investigated the case in which eGFR < 60 ml/min/1.73 m^2^ (stage 3 nephropathy) which has been significantly associated with increased CHD events^23^,^24^. If there is a causal influence of eGFR on CHD, it may be stronger in those for whom an increased association is observed. By conditioning on eGFR, a biased estimate of causal effect may result^25^. However, we observed little evidence of a causal association between eGFR and CHD with the unweighted score (OR = 0.982, 95% CI = 0.963–1.002, p = 0.083) and the weighted score (OR = 0.991, 95% CI = 0.974–1.008, p = 0.286) when restricting analysis to subjects with eGFR < 60.

Since the gene scores were not specific to eGFR but also associated with other kidney function traits, we adjusted the regression of CHD on eGFR for creatinine and serum urea concentration in the UCLEB data alone. Neither gene score was significantly associated with CHD in the UCLEB data, with or without adjustment for creatinine and serum urea concentration.

Finally, the reverse MR did not show an association of the CHD gene score with eGFR (β = −0.120, 95% CI = −1.271–0.872, p = 0.715).

## Discussion

We applied Mendelian Randomisation to determine whether lower eGFR has a causal role in CHD. To improve the power of MR, we combined 17 SNPs into a single gene score. By using a richly phenotyped data set in the UCLEB consortium, we thoroughly examined whether our gene score meets the assumptions of Mendelian Randomisation studies, in that it is associated with eGFR, not associated with potential confounders of the eGFR-CHD association, and not associated with other biomarkers of CHD that might represent alternative pathways than that through eGFR. The gene scores appeared to meet these assumptions although we noted associations with additional kidney function traits that might represent alternative pathways through kidney function to that measured by eGFR.

We found significant evidence of a causal effect on CHD using an unweighted score. However, the weighted score revealed that this result is driven by rs653178, which was not associated with eGFR in UCLEB (and therefore unreliably down-weighted the significance of the unweighted score). Potential reasons that could cause the discrepancy of rs653187 between UCLEB and the previous study by Olden *et al*. are random sampling, low power due to the smaller sample size in UCLEB, and a substantially weaker association between rs653178 and creatinine-based eGFR (p = 1 × 10^−4^) compared to cystatin-C-based eGFR (p = 3.5 × 10^−11^)^22^. Further analyses, including taking the direction of effect from external sources rather than the UCLEB data, and selecting study participants based on low eGFR, did not yield any stronger evidence of a causal effect.

One explanation of our results is that, if a causal effect exists, it may act only through some pathways contributing to measured eGFR, marked by rs653178, whereas other pathways marked by the other 16 SNPs do not have a causal effect. However, rs653178 has been previously shown to be associated with a number of phenotypes, including mean arterial pressure^26^, blood pressure^27^, celiac disease^28^ and peripheral artery disease, a known complication of both CHD and CKD^29^. Therefore the known pleiotropic effect of rs653178 restricts inference of a direct causal effect of eGFR on CHD.

Our study has some limitations. Only 1.5% of the variation in eGFR was explained by the gene scores comprising 17 SNPs. In the largest consortium data available, CARDIoGRAMplusC4D including approximately 200,000 individuals, we would have 74% and 25% power to detect causal odds ratios of 0.9 and 0.95 respectively^30^, both in excess of the observational odds ratio of 0.98 in the UCLEB consortium. Although the study was likely underpowered over the full range of eGFR, a stronger causal effect may exist among those with eGFR < 60, but our gene scores were not associated with CHD among those subjects. A score including more eGFR associated SNPs would have more power to detect a causal effect, though at greater risk of violating the MR assumptions. Another limitation concerns the validation of the MR assumptions for our gene scores. While UCLEB provides a richly phenotyped and large data set, other phenotypes not measured in those studies may be confounding factors or show pleiotropic effects of our gene scores. Furthermore our power to detect confounding and pleiotropy was reduced by control for multiple testing, which we deemed necessary to avoid falsely inferring that our gene scores are invalid instruments.

Observational studies have mainly shown increased risk of CHD among those with low MDRD eGFR compared to those in the normal range. Studies using newer biomarkers provide clear evidence that there is association even at higher eGFR^31^. We have treated eGFR as a continuous variable in MR analysis, which may have reduced power if the causal effect is restricted to subjects with low MDRD eGFR. We took this approach because the associated SNPs apparently influence eGFR over its entire range so that a standard MR analysis would correspond to a population-wide intervention on eGFR levels. Again however, we found no association between the gene scores and CHD among subjects with low eGFR. More robust methods for MR analysis in discrete exposure strata have recently become available^25^,^32^ and could be applied here. However, we believe our current results are not sufficiently encouraging to warrant these approaches.

We are not the first to observe that inflammatory and thrombotic markers associated with CHD are raised in people with CKD^5^. These potential confounding biomarkers may be a result of other life style associated risk factors leading to CKD, in particular overweight at younger age and subsequent obesity^33^,^34^,^35^. In total we identified 27 potential confounders but upon adjustment the association between eGFR and CHD remained significant with a similar odds ratio. Our power to detect confounders was limited by the number of cases of CHD in UCLEB, the range of phenotypes considered (in particular excluding socio-economic status), the limited sample size for some particular phenotypes and the multiple testing burden, and further confounders may exist that attenuate or abolish the eGFR-CHD association. We used reverse MR to address the possibility of reverse causation, but did not observe significant association between a gene score for CHD and levels of eGFR.

In conclusion, this study observed weak evidence for a causal effect of low eGFR on CHD risk, while the observational association was not explained by potential confounders nor by reverse causation. However, this result was highly influenced by rs653178, which has known pleiotropic effects, therefore restricting any inference of a direct causal effect of eGFR on CHD. Our results leave the remaining unexplained association between eGFR and CHD as an open question.

## Additional Information

**How to cite this article**: Charoen, P. *et al*. Mendelian Randomisation study of the influence of eGFR on coronary heart disease. *Sci. Rep.* **6**, 28514; doi: 10.1038/srep28514 (2016).

## Supplementary Material

Supplementary Information

## Figures and Tables

**Table 1:** Descriptive table for CHD cases and controls in 7 studies.

	N (available CHD)		N (available eGFR)		Mean age		% Male		Mean eGFR (ml/min/1.73m^2^)	
	Cases	Controls	Cases	Controls	Cases	Controls	Cases	Controls	Cases	Controls
BRHS	630	1823	619	1802	69.2	68.8	100	100	61.9	63.7
BWHHS	338	1686	330	1647	71.3	70.6	0	0	60.2	63.5
CAPS	354	1040	343	974	57.0	56.7	100	100	69.8	69.2
ET2D	227	830	223	826	69.0	67.6	73.1	45.5	63.6	72.1
EAS	185	670	0	0	70.8	69.7	57.8	46.3	NA	NA
ELSA	316	1669	0	0	75.4	73.3	65.2	50.7	NA	NA
WHII	199	3178	0	0	63.3	60.6	85.4	75.0	NA	NA
