# Supplementary material for: Mendelian Randomisation study of the influence of eGFR on coronary heart disease
Source: Sci Rep. 2016 Jun 24;6:28514. doi: 10.1038/srep28514 (PMC4919785; doi:10.1038/srep28514)
Supplement: Supplementary Information [file srep28514-s1.doc]

**Supplementary Tables and Figures**

**Mendelian Randomisation study of the influence of eGFR on coronary heart disease**

Pimphen Charoen ^1,2^ , Dorothea Nitsch^1^, Jorgen Engmann^3^, Tina Shah^3^, Jonathan White^3^, Delilah Zabaneh^3^, Barbara Jefferis^4^ , Goya Wannamethee^4^, Peter Whincup^5^, Amy Mulick Cassidy^1^, Tom Gaunt^6^, Ian Day^6^, Stela McLachlan^7^, Jacqueline Price^7^, Meena Kumari^3, 8^, Mika Kivimaki^9^, Eric Brunner^9^, Claudia Langenberg^10,11^, Yoav Ben-Shlomo^12^, Aroon Hingorani^3^, John Whittaker^1, 13^, Juan Pablo Casas^1, 3^, Frank Dudbridge^1*^, the UCLEB Consortium

| *GENE(s)* in hit regions | Function (if known) | Marker SNPs |
| --- | --- | --- |
| *NAT8, NAT8B,ALMS1*  *DUSP11, TPRKB* | acetylation enzyme, NAT8 expressed in tubular cells  known monogenetic kidney disease: Alstrom  unknown | rs10206899, rs15358 |
| *DAB2*  *C9* | Proximal tubular adaptor protein, links to megalin  complement C9 locus | rs11959928 |
| *SLC34A1* | Na Phosphate transporter, associated with stones | rs6420094 |
| *DACH1* | involved in organogenesis, tubular epithelial cells | rs626277 |
| *STC1* | Stanniocalcin 1, expressed in human nephron, involved in fish in calcium metabolism | rs1731274 |
| *UMOD* | Tamm-Horsfall protein/Uromodulin, rare variants associated with medullary cystic kidney disease type 2, familial juvenile hyper-uricemic nephropathy, glomerulo-cystic kidney disease, known hypertension gene | rs12917707  rs4293393 rs13333226 |
| *SLC7A9* | amino-acid transporter in tubular cells (including lysine), causes cystinuria, nephrolithiasis | rs4805834  rs8101881 |
| *SOX11* | embryonal transcription factor | rs16864170 |
| *VEGFA* | Vascular endothelial growth factor | rs881858 |
| *PKRAG2* | rare variants associated with WPW, and cardiac changes and hypertrophy of kidney | rs7805747 |
| *ATXN2* | Associated with retinal vessel caliber, celiac disease, diabetes type 1, myocardial infarction, blood pressure variant | rs653178 |
| *TBX2* | T-box transcription factors, no known kidney phenotype | rs8068318 |
| *GCKR, IFT172, FNDC4* | Pleiotropic locus. Common variants in *GCKR* are associated with a variety of human traits in genetic association studies, including serum triglycerides, fasting glucose, C-reactive protein and uric acid as well as susceptibility to type 2 diabetes. | rs1260326 |
| *ANXA9, FAM63A, PRUNE, BNIPL, LASS2, SETDB1* | Unknown | rs267734 |
| *SYPL2, ATXN7L2, CYB561D1, PSMA5, AMIGO1, SORT1* | Unknown | rs1933182 |
| *TFDP2* | Transcription factor involved in cell cycle | rs347685 |
| *SHROOM3* | expressed in kidney, neuro-epithelial development, associated with low blood magnesium in genome-wide association study, and borderline association with UACR | rs17319721  rs9992101 |
| *PIP5K1B* | Unknown | rs4744712 |
| *RNASEH2C, KAT5 OVOL1* | *OVOL1* is zinc-finger transcription factor, downstream of WNT signalling | rs4014195 |
| *UBE2Q2* | belongs to the ubiquitin-conjugating enzyme family, potentially involved in degradation of tubular transporter proteins, potential cancer suppressor gene | rs1394125 |
| *MHC region* | HLA region known to be associated with autoimmune forms of kidney disease | rs3828890 |
| *UNCX* | Associated with blood urea nitrogen in East Asians, encodes a paired-type homeobox transcription factor that has essential roles in skeleton formation and kidney development | rs10277115 |
| *MPPED2-DCDC5* | Unknown  Associated with blood urea nitrogen in East Asians, and with serum magnesium levels | rs963837 |
| *BCAS3* | Unknown  Associated with blood urea nitrogen and uric acid in East Asians | rs9895661 |
| *WDR72* | Unknown  Associated with blood urea nitrogen in East Asians | rs17730281  rs491567 |
| *CUBN* | Cubilin - interacts with megalin and as a receptor for intrinsic factor-vitamin B12 complexes. Associated with low HDL, known CVD risk gene | rs1801239 |

S1 Table. Consistent and replicated SNP markers for CKD in European populations . All markers were associated with eGFR, except *CUBN,* which was associated with an incident urinary albumin/creatinine ratio. SNPs in genes that encode for synthesis or secretion of cystatin C and creatinine (blood markers for eGFR) are excluded.

| snps used in our MR study (17 snps in total) |
| --- |
| snps removed due to high LD |
| snps not available in Metabochip (UCLEB) |

| chr | reported eGFR SNPs | CARDIoGRAMplusC4D | | |
| --- | --- | --- | --- | --- |
|  |  | CARDIoGRAM GWAS  (22,233 CHD cases and 64,762 controls) | C4D GWAS  (15,420 CHD cases and 15,062 controls) | CARDIoGRAMplusC4D Metabochip   (63,746 CHD cases and 130,681 controls) |
| 1 | rs267734 | y |  | y |
| 1 | rs1933182 | y |  |  |
| 2 | rs10206899 | y |  |  |
| 2 | rs16864170 |  |  |  |
| 2 | rs1260326 | y | y |  |
| 3 | rs347685 |  |  |  |
| 4 | rs17319721 | y |  |  |
| 4 | rs9992101 |  |  |  |
| 5 | rs11959928 |  |  |  |
| 5 | rs6420094 | y | y |  |
| 6 | rs881858 | y |  |  |
| 6 | rs3828890 |  |  |  |
| 7 | rs7805747 | y | y |  |
| 7 | rs10277115 |  |  |  |
| 8 | rs1731274 |  |  |  |
| 9 | rs4744712 | y |  | y |
| 10 | rs1801239 |  |  |  |
| 11 | rs4014195 | y |  |  |
| 11 | rs963837 |  |  |  |
| 12 | rs653178 | y | y | y |
| 13 | rs15358 |  |  |  |
| 13 | rs626277 |  |  |  |
| 15 | rs1394125 |  |  |  |
| 15 | rs17730281 | y |  |  |
| 15 | rs491567 |  |  |  |
| 16 | rs12917707 | y |  |  |
| 16 | rs4293393 |  |  |  |
| 16 | rs13333226 |  |  |  |
| 17 | rs8068318 | y |  |  |
| 17 | rs9895661 |  |  |  |
| 19 | rs4805834 |  |  |  |
| 19 | rs8101881 | y | y | y |
|  |  |  |  |  |

S2 Table. Selection of 17 SNPs and their availability in CARDIoGRAMplusC4D. Out of 32 SNPs listed in Supplementary Table I, 11 SNPs (brown boxes) are not available in UCLEB. A further 4 SNPs (light blue boxes) are shown as high LD SNPs (r^2^>0.7). Hence the 17 remaining SNPs (dark blue box) are selected as potential instrumental variables. “y” represents the availability of SNPs across 3 data sets from CARDIoGRAMplusC4D.

| Abbreviation | Description |
| --- | --- |
| sex | sex |
| age | age |
| whr | Waist to hip ratio |
| wcir | Waist circumference |
| bmi | Body mass index |
| pbfat | % body fat |
| ht | height |
| wt | Weight |
| sbp | Systolic blood pressure |
| dbp | Diastolic blood pressure |
| smoke | smoking (ever/never) |
| alcohol | Alcohol |
| Tc | TC |
| ldl | LDL |
| hdl | HDL |
| tg | Triglyceride |
| lpa | Lp(a) |
| hba1c | HbA1c |
| glucose | glucose |
| insulin | insulin |
| viscosity | Plasma viscosity |
| hct | Haematocrit |
| fact8 | Factor VIII |
| fact9 | FactorIX |
| aptt | Activated partial thromboplastin time |
| tpa | tPa |
| vwf | vW factor |
| ddimer | D-Dimer |
| fact7 | Factor VII |
| wcc | WCC |
| fib | Fib |
| il6 | IL-6 |
| tnfalpha | TNF-alpha |
| platelet | Platelets |
| rbc | Red blood cell count |
| haemoglobin | Heamoglobin levels |
| mcv | Mean cell volume |
| mch | Mean call haemoglobin |
| mpv | Mean platelet count |
| neutrophil | Neutrophil |
| lympho | Lymphocytes |
| mono | Monocytes |
| eosinophil | Eosinophils |
| basinophil | Basinophils |
| mchc | Mean cellular Haemoglobin |
| crp | CRP |
| ferr | Ferritin |
| ggt | GGT |
| alt | ALT |
| alkphos | Alk Phos |
| albumin | Albumin |
| bilirubin | Bilirubin |
| ast | AST |
| creatinine | Creatinine |
| surea | Serum urea concentration |
| spotassium | Serum potassium concentration |
| ssodium | Serum sodium concentration |
| surate | Serum urate concentration |
| smagnesium | Serum magnesium concentration |
| scalcium | Serum calcium concentration |
| ccalcium | Corrected calcium concentration |
| sphosph | Serum phosphate concentration |
| sprotein | Total serum protein concentration |
| egfr | eGFR |
| pwave | ECG P wave |
| printerval | ECG PR interval |
| qtinterval | ECG QT interval |
| qtc | ECG QTc |
| qrsvoltsum | ECG QRS voltage sum |
| qrsvoltprod | ECG QRS voltage product |
| cornellprod | ECG Cornell product |
| sokolowlyon | ECG Sokolow Lyon |
| sclass | Social class |
| occup | Occupation |
| fev1 | FEV1 |
| fvc | FVC |
| pefr | Peak Expiratory Flow Rate |
| probnp | NT-proBNP |
| leptin | Leptin |
| igf1 | IGF-1 |
| vitc | Vitamin C |
| vite | Vitamin E |
| betacarot | Beta-carotene |
| adiponectin | Adiponectin |
| il18 | IL-18 |
| mmp9 | MMP-9 |
| scd401 | sCD40L |
| cotinine | Cotinine |

S3 Table. Phenotype descriptions in UCLEB

|  | eGFR-trait | | CHD-trait | | unweighted gene score-trait | | internally-weighted gene score-trait | |
| --- | --- | --- | --- | --- | --- | --- | --- | --- |
|  | unadjusted | adjusted | unadjusted | adjusted | unadjusted | adjusted | unadjusted | adjusted |
| adiponectin | 9.56E-01 | 9.57E-01 | 6.20E-01 | 6.89E-01 | 1.82E-02 | 3.57E-01 | 1.31E-02 | 2.56E-01 |
| age* | 8.45E-58 | 6.93E-56 | 1.05E-14 | 2.41E-13 | 8.77E-01 | 9.94E-01 | 8.12E-01 | 9.77E-01 |
| albumin | 1.65E-08 | 5.90E-08 | 4.22E-01 | 4.98E-01 | 2.99E-01 | 9.05E-01 | 4.94E-01 | 8.86E-01 |
| alcohol* | 7.91E-05 | 1.85E-04 | 1.46E-02 | 2.80E-02 | 5.23E-01 | 9.94E-01 | 2.36E-01 | 8.51E-01 |
| alkphos | 1.15E-02 | 2.20E-02 | 9.46E-02 | 1.45E-01 | 6.28E-01 | 9.94E-01 | 6.06E-01 | 9.25E-01 |
| alt | 1.78E-10 | 9.13E-10 | 7.31E-01 | 7.78E-01 | 5.73E-01 | 9.94E-01 | 9.54E-01 | 9.77E-01 |
| apoa1 | NA | NA | 9.13E-06 | 3.50E-05 | 2.23E-01 | 9.05E-01 | 1.66E-01 | 7.63E-01 |
| apob | NA | NA | 2.49E-13 | 3.27E-12 | 8.79E-01 | 9.94E-01 | 1.12E-01 | 7.63E-01 |
| apoe | NA | NA | 2.31E-06 | 1.01E-05 | 9.54E-02 | 8.50E-01 | 2.72E-01 | 8.62E-01 |
| aptt | 9.56E-01 | 9.57E-01 | 7.38E-02 | 1.17E-01 | 7.44E-01 | 9.94E-01 | 3.87E-01 | 8.86E-01 |
| ast | 2.71E-02 | 4.63E-02 | 6.62E-01 | 7.25E-01 | 6.49E-01 | 9.94E-01 | 3.73E-01 | 8.86E-01 |
| basinophil | 6.03E-03 | 1.18E-02 | 1.31E-01 | 1.95E-01 | 8.72E-01 | 9.94E-01 | 8.54E-01 | 9.77E-01 |
| betacarot | 7.04E-01 | 7.49E-01 | 2.28E-02 | 4.11E-02 | 9.99E-01 | 9.99E-01 | 7.17E-01 | 9.77E-01 |
| bilirubin | 3.67E-01 | 4.55E-01 | 6.01E-02 | 9.88E-02 | 2.80E-01 | 9.05E-01 | 2.90E-01 | 8.62E-01 |
| bmi* | 1.26E-17 | 1.72E-16 | 7.28E-06 | 2.91E-05 | 8.77E-01 | 9.94E-01 | 3.56E-01 | 8.86E-01 |
| ccalcium | 1.38E-08 | 5.13E-08 | 1.49E-01 | 2.17E-01 | 1.80E-01 | 9.05E-01 | 1.81E-01 | 7.63E-01 |
| cornellprod* | 1.10E-03 | 2.38E-03 | 4.31E-07 | 2.09E-06 | 7.66E-01 | 9.94E-01 | 6.67E-01 | 9.76E-01 |
| cotinine* | 4.81E-03 | 9.62E-03 | 1.38E-04 | 3.63E-04 | 4.30E-01 | 9.65E-01 | 1.09E-01 | 7.63E-01 |
| creatinine(**) | - | - | - | - | 1.23E-10 | 6.02E-09 | 1.39E-17 | 6.83E-16 |
| crp* | 4.29E-09 | 1.85E-08 | 1.56E-05 | 5.51E-05 | 8.56E-01 | 9.94E-01 | 1.58E-01 | 7.63E-01 |
| dbp | 2.80E-01 | 3.58E-01 | 3.32E-03 | 7.11E-03 | 9.75E-01 | 9.97E-01 | 6.02E-01 | 9.25E-01 |
| ddimer* | 6.19E-16 | 6.35E-15 | 3.08E-05 | 1.01E-04 | 6.58E-01 | 9.94E-01 | 4.10E-01 | 8.86E-01 |
| egfr(**) | - | - | - | - | 2.01E-14 | 1.97E-12 | 3.37E-21 | 3.30E-19 |
| eosinophil* | 3.85E-12 | 3.16E-11 | 1.87E-03 | 4.42E-03 | 4.30E-01 | 9.65E-01 | 1.87E-01 | 7.63E-01 |
| esel | NA | NA | 1.73E-01 | 2.45E-01 | 4.35E-01 | 9.65E-01 | 4.13E-01 | 8.86E-01 |
| fact7 | 1.94E-11 | 1.33E-10 | 2.08E-01 | 2.89E-01 | 8.79E-01 | 9.94E-01 | 9.35E-01 | 9.77E-01 |
| fact8* | 1.61E-20 | 3.30E-19 | 1.77E-09 | 1.36E-08 | 1.54E-01 | 9.05E-01 | 8.73E-01 | 9.77E-01 |
| fact9* | 1.24E-08 | 4.85E-08 | 3.94E-03 | 8.23E-03 | 3.47E-02 | 4.25E-01 | 1.17E-01 | 7.63E-01 |
| ferr | NA | NA | 3.11E-01 | 3.93E-01 | 4.43E-01 | 9.65E-01 | 5.88E-01 | 9.25E-01 |
| fev1* | 6.78E-05 | 1.64E-04 | 1.82E-11 | 1.67E-10 | 4.93E-01 | 9.94E-01 | 6.76E-02 | 7.63E-01 |
| fib* | 4.99E-17 | 5.84E-16 | 2.24E-13 | 3.27E-12 | 2.36E-01 | 9.05E-01 | 4.39E-01 | 8.86E-01 |
| fvc* | 1.63E-06 | 4.61E-06 | 1.97E-12 | 2.02E-11 | 2.69E-01 | 9.05E-01 | 1.94E-02 | 3.16E-01 |
| ggt | 3.41E-02 | 5.60E-02 | 1.19E-01 | 1.79E-01 | 5.47E-01 | 9.94E-01 | 5.25E-01 | 8.86E-01 |
| glucose | 4.39E-01 | 5.37E-01 | 1.65E-06 | 7.61E-06 | 3.29E-02 | 4.25E-01 | 5.40E-01 | 8.86E-01 |
| haemoglobin | 4.51E-19 | 7.40E-18 | 3.12E-01 | 3.93E-01 | 9.50E-01 | 9.97E-01 | 5.15E-01 | 8.86E-01 |
| hba1c | 5.89E-01 | 6.70E-01 | 4.18E-08 | 2.41E-07 | 3.04E-01 | 9.05E-01 | 9.76E-01 | 9.86E-01 |
| hct | 3.38E-36 | 1.39E-34 | 4.13E-01 | 4.98E-01 | 9.84E-01 | 9.97E-01 | 9.26E-01 | 9.77E-01 |
| hdl* | 1.10E-10 | 6.02E-10 | 2.27E-39 | 2.09E-37 | 5.71E-01 | 9.94E-01 | 9.54E-01 | 9.77E-01 |
| ht | 1.59E-02 | 2.77E-02 | 7.75E-01 | 7.92E-01 | 2.89E-03 | 7.09E-02 | 4.81E-03 | 1.18E-01 |
| icam | NA | NA | 5.27E-01 | 6.06E-01 | 7.10E-01 | 9.94E-01 | 7.33E-01 | 9.77E-01 |
| igf1 | 6.73E-01 | 7.26E-01 | 6.02E-01 | 6.84E-01 | 2.90E-01 | 9.05E-01 | 8.65E-01 | 9.77E-01 |
| il18 | 7.76E-01 | 8.06E-01 | 6.21E-01 | 6.89E-01 | 7.80E-02 | 7.64E-01 | 9.11E-02 | 7.63E-01 |
| il6* | 6.93E-06 | 1.89E-05 | 3.24E-05 | 1.03E-04 | 4.95E-01 | 9.94E-01 | 9.57E-01 | 9.77E-01 |
| insulin | 5.85E-01 | 6.70E-01 | 6.09E-03 | 1.22E-02 | 1.69E-01 | 9.05E-01 | 4.50E-01 | 8.86E-01 |
| ldl | 1.53E-02 | 2.74E-02 | 6.00E-02 | 9.88E-02 | 1.10E-01 | 9.02E-01 | 7.42E-01 | 9.77E-01 |
| leptin | 1.03E-04 | 2.35E-04 | 8.57E-01 | 8.57E-01 | 9.51E-01 | 9.97E-01 | 9.11E-01 | 9.77E-01 |
| lpa | NA | NA | 4.07E-05 | 1.25E-04 | 3.20E-02 | 4.25E-01 | 1.56E-01 | 7.63E-01 |
| lympho | 3.43E-01 | 4.33E-01 | 7.29E-01 | 7.78E-01 | 3.11E-01 | 9.05E-01 | 3.55E-01 | 8.86E-01 |
| mch | 1.47E-02 | 2.68E-02 | 3.98E-02 | 7.04E-02 | 8.56E-01 | 9.94E-01 | 1.54E-01 | 7.63E-01 |
| mchc | 1.19E-02 | 2.22E-02 | 7.36E-01 | 7.78E-01 | 8.34E-01 | 9.94E-01 | 7.65E-01 | 9.77E-01 |
| mcv | 6.29E-01 | 7.06E-01 | 4.40E-01 | 5.12E-01 | 6.94E-01 | 9.94E-01 | 1.42E-01 | 7.63E-01 |
| mmp9 | 2.46E-01 | 3.31E-01 | 1.26E-03 | 3.06E-03 | 3.05E-01 | 9.05E-01 | 1.64E-01 | 7.63E-01 |
| mono | 7.75E-02 | 1.16E-01 | 2.77E-08 | 1.70E-07 | 3.53E-01 | 9.50E-01 | 2.59E-01 | 8.62E-01 |
| mpv | 1.12E-01 | 1.61E-01 | 2.97E-03 | 6.67E-03 | 5.84E-01 | 9.94E-01 | 5.27E-01 | 8.86E-01 |
| neutrophil* | 2.50E-05 | 6.42E-05 | 1.43E-14 | 2.63E-13 | 9.01E-01 | 9.97E-01 | 8.93E-01 | 9.77E-01 |
| occup | 2.61E-07 | 7.92E-07 | 7.61E-02 | 1.19E-01 | 3.59E-01 | 9.50E-01 | 8.30E-01 | 9.77E-01 |
| pbfat* | 5.41E-05 | 1.34E-04 | 4.52E-05 | 1.34E-04 | 8.77E-01 | 9.94E-01 | 7.13E-01 | 9.77E-01 |
| pefr* | 1.32E-04 | 2.92E-04 | 1.54E-05 | 5.51E-05 | 8.80E-01 | 9.94E-01 | 2.19E-01 | 8.27E-01 |
| platelet | 1.51E-05 | 3.99E-05 | 1.72E-01 | 2.45E-01 | 1.76E-01 | 9.05E-01 | 8.40E-01 | 9.77E-01 |
| printerval | 2.88E-03 | 5.90E-03 | 5.34E-02 | 9.09E-02 | 8.36E-01 | 9.94E-01 | 9.33E-01 | 9.77E-01 |
| probnp* | 2.99E-08 | 1.02E-07 | 2.44E-09 | 1.73E-08 | 5.48E-01 | 9.94E-01 | 9.93E-01 | 9.93E-01 |
| prothrombin | NA | NA | 7.69E-01 | 7.92E-01 | 9.62E-01 | 9.97E-01 | 7.82E-01 | 9.77E-01 |
| pwave | 9.57E-01 | 9.57E-01 | 4.20E-01 | 4.98E-01 | 6.58E-01 | 9.94E-01 | 6.14E-01 | 9.25E-01 |
| qrsvoltprod | 7.72E-01 | 8.06E-01 | 2.87E-04 | 7.33E-04 | 7.51E-01 | 9.94E-01 | 8.87E-01 | 9.77E-01 |
| qrsvoltsum | 2.73E-01 | 3.58E-01 | 1.50E-02 | 2.81E-02 | 9.66E-01 | 9.97E-01 | 8.95E-01 | 9.77E-01 |
| qtc* | 4.89E-13 | 4.46E-12 | 4.35E-09 | 2.86E-08 | 2.44E-01 | 9.05E-01 | 1.58E-01 | 7.63E-01 |
| qtinterval* | 5.46E-08 | 1.79E-07 | 8.32E-05 | 2.25E-04 | 3.23E-01 | 9.05E-01 | 5.30E-01 | 8.86E-01 |
| rbc | 2.53E-11 | 1.60E-10 | 3.07E-01 | 3.93E-01 | 7.94E-01 | 9.94E-01 | 8.74E-01 | 9.77E-01 |
| sbp | 2.77E-01 | 3.58E-01 | 6.81E-06 | 2.85E-05 | 9.06E-01 | 9.97E-01 | 5.05E-01 | 8.86E-01 |
| scalcium | 2.34E-01 | 3.20E-01 | 3.86E-01 | 4.80E-01 | 9.54E-01 | 9.97E-01 | 8.29E-01 | 9.77E-01 |
| scd40l | 6.52E-01 | 7.23E-01 | 3.09E-03 | 6.78E-03 | 3.19E-01 | 9.05E-01 | 4.51E-01 | 8.86E-01 |
| sclass | 6.66E-02 | 1.05E-01 | 5.09E-03 | 1.04E-02 | 4.36E-01 | 9.65E-01 | 2.01E-01 | 7.87E-01 |
| sex* | 9.24E-09 | 3.79E-08 | 1.04E-19 | 3.18E-18 | 8.82E-01 | 9.94E-01 | 4.73E-01 | 8.86E-01 |
| smagnesium | 6.99E-02 | 1.06E-01 | 2.32E-01 | 3.19E-01 | 7.56E-01 | 9.94E-01 | 7.53E-01 | 9.77E-01 |
| smoke | 1.41E-01 | 1.96E-01 | 5.08E-08 | 2.75E-07 | 6.94E-01 | 9.94E-01 | 7.84E-01 | 9.77E-01 |
| sokolowlyon | 9.53E-02 | 1.40E-01 | 7.38E-05 | 2.06E-04 | 2.47E-01 | 9.05E-01 | 5.26E-01 | 8.86E-01 |
| sphosph | 4.46E-01 | 5.38E-01 | 2.59E-01 | 3.45E-01 | 4.34E-01 | 9.65E-01 | 2.43E-01 | 8.51E-01 |
| spotassium | 5.16E-02 | 8.30E-02 | 2.46E-01 | 3.32E-01 | 1.88E-01 | 9.05E-01 | 3.84E-01 | 8.86E-01 |
| sprotein* | 1.47E-07 | 4.63E-07 | 6.71E-03 | 1.31E-02 | 2.33E-01 | 9.05E-01 | 2.79E-01 | 8.62E-01 |
| ssodium | 6.70E-01 | 7.26E-01 | 7.47E-01 | 7.81E-01 | 7.39E-01 | 9.94E-01 | 6.67E-01 | 9.76E-01 |
| surate(**) | - | - | - | - | 9.87E-01 | 9.97E-01 | 1.71E-01 | 7.63E-01 |
| surea(**) | - | - | - | - | 3.00E-04 | 9.79E-03 | 9.55E-07 | 3.12E-05 |
| tc | 3.19E-02 | 5.35E-02 | 4.20E-01 | 4.98E-01 | 7.96E-01 | 9.94E-01 | 3.54E-01 | 8.86E-01 |
| tg* | 1.95E-03 | 4.10E-03 | 3.83E-11 | 3.20E-10 | 5.01E-02 | 5.45E-01 | 4.34E-02 | 6.08E-01 |
| tnfalpha | 1.69E-09 | 7.70E-09 | 6.33E-02 | 1.02E-01 | 7.55E-01 | 9.94E-01 | 4.85E-01 | 8.86E-01 |
| tpa* | 1.54E-11 | 1.15E-10 | 2.35E-05 | 8.01E-05 | 7.60E-01 | 9.94E-01 | 5.43E-01 | 8.86E-01 |
| uric | NA | NA | 2.43E-03 | 5.60E-03 | 8.07E-01 | 9.94E-01 | 7.88E-01 | 9.77E-01 |
| vcam | NA | NA | 8.12E-01 | 8.21E-01 | 3.15E-01 | 9.05E-01 | 1.67E-01 | 7.63E-01 |
| viscosity | 6.97E-02 | 1.06E-01 | 5.45E-04 | 1.36E-03 | 6.28E-01 | 9.94E-01 | 5.64E-01 | 9.07E-01 |
| vitc | 5.33E-07 | 1.56E-06 | 4.47E-02 | 7.75E-02 | 5.21E-01 | 9.94E-01 | 5.09E-01 | 8.86E-01 |
| vite | 5.67E-01 | 6.65E-01 | 2.99E-01 | 3.93E-01 | 2.21E-01 | 9.05E-01 | 1.59E-01 | 7.63E-01 |
| vwf* | 8.22E-25 | 2.25E-23 | 9.24E-08 | 4.72E-07 | 4.41E-01 | 9.65E-01 | 8.89E-01 | 9.77E-01 |
| wcc | 5.37E-01 | 6.38E-01 | 1.90E-02 | 3.49E-02 | 1.99E-01 | 9.05E-01 | 3.29E-01 | 8.86E-01 |
| wcir* | 3.49E-10 | 1.69E-09 | 7.11E-13 | 8.17E-12 | 5.75E-01 | 9.94E-01 | 7.29E-01 | 9.77E-01 |
| whr | 1.19E-01 | 1.68E-01 | 2.76E-22 | 1.27E-20 | 4.84E-01 | 9.94E-01 | 3.91E-01 | 8.86E-01 |
| wt* | 6.34E-11 | 3.71E-10 | 5.23E-05 | 1.50E-04 | 1.20E-01 | 9.03E-01 | 4.12E-01 | 8.86E-01 |

S4 Table. Unadjusted and Benjamini-Hochberg adjusted *P*-values for association of each trait with eGFR, CHD, unweighted gene score, and UCLEB internally weighted gene score. Twenty one traits (*) are potential confounding factors. NAs shown in eGFR-trait column are due to missing trait measurement on the samples with available eGFR. Kidney related phenotypes (**) are not considered as potential confounders, so eGFR-trait and CHD-trait associations are not calculated for those phenotypes.

|  | unweighted gene score-trait associations | | | weighted score-trait  associations | | |
| --- | --- | --- | --- | --- | --- | --- |
|  | beta | se | p-value | beta | se | p-value |
| adiponectin | -4.62E-01 | 1.96E-01 | 1.82E-02 | -7.13E-01 | 2.87E-01 | 1.31E-02 |
| age | -2.99E-03 | 1.93E-02 | 8.77E-01 | -7.08E-03 | 2.97E-02 | 8.12E-01 |
| albumin | -1.51E-02 | 1.45E-02 | 2.99E-01 | -1.53E-02 | 2.24E-02 | 4.94E-01 |
| alcohol | -7.38E-03 | 1.16E-02 | 5.23E-01 | -2.09E-02 | 1.76E-02 | 2.36E-01 |
| alkphos | 1.71E-01 | 3.53E-01 | 6.28E-01 | 2.67E-01 | 5.17E-01 | 6.06E-01 |
| alt | -3.42E-02 | 6.07E-02 | 5.73E-01 | -5.40E-03 | 9.27E-02 | 9.54E-01 |
| apoa1 | -3.09E-03 | 2.54E-03 | 2.23E-01 | -5.10E-03 | 3.69E-03 | 1.66E-01 |
| apob | -3.28E-04 | 2.14E-03 | 8.79E-01 | 4.95E-03 | 3.12E-03 | 1.12E-01 |
| apoe | 2.16E-04 | 1.30E-04 | 9.54E-02 | 2.10E-04 | 1.91E-04 | 2.72E-01 |
| aptt | 1.47E-02 | 4.51E-02 | 7.44E-01 | 5.83E-02 | 6.73E-02 | 3.87E-01 |
| ast | -2.87E-02 | 6.31E-02 | 6.49E-01 | -8.57E-02 | 9.62E-02 | 3.73E-01 |
| basinophil | -8.95E-05 | 5.57E-04 | 8.72E-01 | -1.52E-04 | 8.26E-04 | 8.54E-01 |
| betacarot | 1.81E-05 | 2.32E-02 | 9.99E-01 | -1.25E-02 | 3.45E-02 | 7.17E-01 |
| bilirubin | -4.52E-02 | 4.18E-02 | 2.80E-01 | -6.41E-02 | 6.06E-02 | 2.90E-01 |
| bmi | 2.46E-03 | 1.59E-02 | 8.77E-01 | 2.23E-02 | 2.42E-02 | 3.56E-01 |
| ccalcium | 1.19E-03 | 8.92E-04 | 1.80E-01 | 1.78E-03 | 1.33E-03 | 1.81E-01 |
| cornellprod | 1.28E-01 | 4.28E-01 | 7.66E-01 | -2.76E-01 | 6.41E-01 | 6.67E-01 |
| cotinine | 6.71E-01 | 8.51E-01 | 4.30E-01 | 2.03E+00 | 1.27E+00 | 1.09E-01 |
| creatinine | -5.52E-01 | 8.57E-02 | 1.23E-10 | -1.08E+00 | 1.27E-01 | 1.39E-17 |
| crp | -5.05E-03 | 2.78E-02 | 8.56E-01 | -5.87E-02 | 4.16E-02 | 1.58E-01 |
| dbp | 1.28E-03 | 4.12E-02 | 9.75E-01 | -3.23E-02 | 6.19E-02 | 6.02E-01 |
| ddimer | -5.24E-01 | 1.18E+00 | 6.58E-01 | -1.49E+00 | 1.80E+00 | 4.10E-01 |
| egfr | 4.97E-01 | 6.50E-02 | 2.01E-14 | 9.54E-01 | 1.01E-01 | 3.37E-21 |
| eosinophil | 9.49E-04 | 1.20E-03 | 4.30E-01 | -2.35E-03 | 1.78E-03 | 1.87E-01 |
| esel | -2.41E-01 | 3.08E-01 | 4.35E-01 | -3.72E-01 | 4.54E-01 | 4.13E-01 |
| fact7 | 2.22E-02 | 1.46E-01 | 8.79E-01 | -1.81E-02 | 2.21E-01 | 9.35E-01 |
| fact8 | 3.18E-01 | 2.23E-01 | 1.54E-01 | 5.52E-02 | 3.47E-01 | 8.73E-01 |
| fact9 | 5.43E-01 | 2.57E-01 | 3.47E-02 | 6.01E-01 | 3.84E-01 | 1.17E-01 |
| ferr | 5.12E-01 | 6.68E-01 | 4.43E-01 | 5.61E-01 | 1.04E+00 | 5.88E-01 |
| fev1 | -2.13E-03 | 3.11E-03 | 4.93E-01 | -8.77E-03 | 4.80E-03 | 6.76E-02 |
| fib | 3.30E-03 | 2.78E-03 | 2.36E-01 | 3.21E-03 | 4.16E-03 | 4.39E-01 |
| fvc | -4.51E-03 | 4.08E-03 | 2.69E-01 | -1.48E-02 | 6.33E-03 | 1.94E-02 |
| ggt | 1.65E-01 | 2.75E-01 | 5.47E-01 | 2.59E-01 | 4.08E-01 | 5.25E-01 |
| glucose | -1.07E-02 | 5.04E-03 | 3.29E-02 | -4.66E-03 | 7.60E-03 | 5.40E-01 |
| haemoglobin | -9.00E-04 | 1.42E-02 | 9.50E-01 | -1.20E-02 | 1.84E-02 | 5.15E-01 |
| hba1c | -5.47E-03 | 5.32E-03 | 3.04E-01 | 2.33E-04 | 7.87E-03 | 9.76E-01 |
| hct | -1.78E-04 | 8.66E-03 | 9.84E-01 | -1.27E-03 | 1.36E-02 | 9.26E-01 |
| hdl | -7.76E-04 | 1.37E-03 | 5.71E-01 | -1.21E-04 | 2.08E-03 | 9.54E-01 |
| ht | -8.91E-04 | 2.99E-04 | 2.89E-03 | -1.28E-03 | 4.54E-04 | 4.81E-03 |
| icam | -4.67E-01 | 1.26E+00 | 7.10E-01 | -6.31E-01 | 1.85E+00 | 7.33E-01 |
| igf1 | -9.72E-01 | 9.18E-01 | 2.90E-01 | -2.29E-01 | 1.35E+00 | 8.65E-01 |
| il18 | 1.49E+01 | 8.43E+00 | 7.80E-02 | 2.10E+01 | 1.24E+01 | 9.11E-02 |
| il6 | 9.51E-03 | 1.39E-02 | 4.95E-01 | 1.12E-03 | 2.09E-02 | 9.57E-01 |
| insulin | -2.34E-01 | 1.70E-01 | 1.69E-01 | -1.17E-01 | 1.55E-01 | 4.50E-01 |
| ldl | -5.97E-03 | 3.74E-03 | 1.10E-01 | -1.88E-03 | 5.70E-03 | 7.42E-01 |
| leptin | 2.93E-02 | 4.79E-01 | 9.51E-01 | -7.88E-02 | 7.03E-01 | 9.11E-01 |
| lpa | 4.31E-01 | 2.01E-01 | 3.20E-02 | 4.15E-01 | 2.93E-01 | 1.56E-01 |
| lympho | 3.93E-02 | 3.88E-02 | 3.11E-01 | 5.32E-02 | 5.75E-02 | 3.55E-01 |
| mch | -1.84E-03 | 1.01E-02 | 8.56E-01 | -2.25E-02 | 1.58E-02 | 1.54E-01 |
| mchc | 2.32E-03 | 1.11E-02 | 8.34E-01 | -4.18E-03 | 1.40E-02 | 7.65E-01 |
| mcv | -1.02E-02 | 2.59E-02 | 6.94E-01 | -5.86E-02 | 3.99E-02 | 1.42E-01 |
| mmp9 | 3.88E+00 | 3.78E+00 | 3.05E-01 | 7.81E+00 | 5.61E+00 | 1.64E-01 |
| mono | 1.12E-03 | 1.20E-03 | 3.53E-01 | 2.02E-03 | 1.79E-03 | 2.59E-01 |
| mpv | 5.53E-03 | 1.01E-02 | 5.84E-01 | 9.81E-03 | 1.55E-02 | 5.27E-01 |
| neutrophil | -1.46E-03 | 1.17E-02 | 9.01E-01 | -2.34E-03 | 1.73E-02 | 8.93E-01 |
| occup | 3.13E-03 | 3.41E-03 | 3.59E-01 | 1.13E-03 | 5.29E-03 | 8.30E-01 |
| pbfat | 1.51E-02 | 9.71E-02 | 8.77E-01 | 5.23E-02 | 1.42E-01 | 7.13E-01 |
| pefr | 8.49E-02 | 5.63E-01 | 8.80E-01 | -1.07E+00 | 8.68E-01 | 2.19E-01 |
| platelet | 4.88E-01 | 3.61E-01 | 1.76E-01 | 1.12E-01 | 5.58E-01 | 8.40E-01 |
| printerval | -2.65E-02 | 1.28E-01 | 8.36E-01 | 1.58E-02 | 1.90E-01 | 9.33E-01 |
| probnp | 3.64E+00 | 6.05E+00 | 5.48E-01 | -7.65E-02 | 8.99E+00 | 9.93E-01 |
| prothrombin | -4.77E-08 | 1.01E-06 | 9.62E-01 | -4.15E-07 | 1.50E-06 | 7.82E-01 |
| pwave | -6.90E-02 | 1.56E-01 | 6.58E-01 | -9.88E-02 | 1.96E-01 | 6.14E-01 |
| qrsvoltprod | -9.82E-01 | 3.10E+00 | 7.51E-01 | -6.65E-01 | 4.67E+00 | 8.87E-01 |
| qrsvoltsum | -9.13E-01 | 2.13E+01 | 9.66E-01 | 4.26E+00 | 3.22E+01 | 8.95E-01 |
| qtc | -1.54E-01 | 1.32E-01 | 2.44E-01 | -2.79E-01 | 1.97E-01 | 1.58E-01 |
| qtinterval | -1.78E-01 | 1.80E-01 | 3.23E-01 | -1.70E-01 | 2.70E-01 | 5.30E-01 |
| rbc | -5.47E-04 | 2.09E-03 | 7.94E-01 | 5.14E-04 | 3.24E-03 | 8.74E-01 |
| sbp | 8.36E-03 | 7.06E-02 | 9.06E-01 | 7.12E-02 | 1.07E-01 | 5.05E-01 |
| scalcium | -4.31E-04 | 7.41E-03 | 9.54E-01 | -8.43E-04 | 3.91E-03 | 8.29E-01 |
| scd40l | 3.86E-02 | 3.87E-02 | 3.19E-01 | 4.33E-02 | 5.75E-02 | 4.51E-01 |
| sclass | -4.40E-03 | 5.65E-03 | 4.36E-01 | -1.11E-02 | 8.70E-03 | 2.01E-01 |
| sex | 1.33E-03 | 9.00E-03 | 8.82E-01 | 9.70E-03 | 1.35E-02 | 4.73E-01 |
| smagnesium | -1.63E-03 | 5.23E-03 | 7.56E-01 | -8.55E-04 | 2.72E-03 | 7.53E-01 |
| smoke | 2.94E-03 | 7.49E-03 | 6.94E-01 | -3.11E-03 | 1.13E-02 | 7.84E-01 |
| sokolowlyon | -4.03E+00 | 3.48E+00 | 2.47E-01 | -3.30E+00 | 5.21E+00 | 5.26E-01 |
| sphosph | 1.18E-03 | 1.50E-03 | 4.34E-01 | 2.62E-03 | 2.24E-03 | 2.43E-01 |
| spotassium | -2.87E-02 | 2.18E-02 | 1.88E-01 | -1.12E-02 | 1.29E-02 | 3.84E-01 |
| sprotein | 3.55E-02 | 2.98E-02 | 2.33E-01 | 5.00E-02 | 4.61E-02 | 2.79E-01 |
| ssodium | 6.21E-02 | 1.87E-01 | 7.39E-01 | 5.04E-02 | 1.17E-01 | 6.67E-01 |
| surate | 9.11E-06 | 5.48E-04 | 9.87E-01 | -1.16E-03 | 8.50E-04 | 1.71E-01 |
| surea | -3.39E-02 | 9.37E-03 | 3.00E-04 | -7.16E-02 | 1.46E-02 | 9.55E-07 |
| tc | -1.00E-03 | 3.89E-03 | 7.96E-01 | 5.48E-03 | 5.92E-03 | 3.54E-01 |
| tg | 8.58E-03 | 4.38E-03 | 5.01E-02 | 1.33E-02 | 6.58E-03 | 4.34E-02 |
| tnfalpha | -4.97E-03 | 1.60E-02 | 7.55E-01 | -1.64E-02 | 2.34E-02 | 4.85E-01 |
| tpa | -8.32E-03 | 2.73E-02 | 7.60E-01 | 2.56E-02 | 4.21E-02 | 5.43E-01 |
| uric | 4.44E-03 | 1.82E-02 | 8.07E-01 | 7.24E-03 | 2.70E-02 | 7.88E-01 |
| vcam | -1.82E+00 | 1.81E+00 | 3.15E-01 | -3.68E+00 | 2.67E+00 | 1.67E-01 |
| viscosity | -1.32E-03 | 2.73E-03 | 6.28E-01 | -8.35E-04 | 1.45E-03 | 5.64E-01 |
| vitc | 1.74E-01 | 2.72E-01 | 5.21E-01 | 2.68E-01 | 4.05E-01 | 5.09E-01 |
| vite | 1.74E-01 | 1.42E-01 | 2.21E-01 | 2.98E-01 | 2.12E-01 | 1.59E-01 |
| vwf | 1.65E-01 | 2.14E-01 | 4.41E-01 | -4.49E-02 | 3.22E-01 | 8.89E-01 |
| wcc | 2.74E-02 | 2.14E-02 | 1.99E-01 | 3.27E-02 | 3.35E-02 | 3.29E-01 |
| wcir | -2.44E-02 | 4.36E-02 | 5.75E-01 | 2.29E-02 | 6.62E-02 | 7.29E-01 |
| whr | 2.02E-04 | 2.88E-04 | 4.84E-01 | 3.74E-04 | 4.37E-04 | 3.91E-01 |
| wt | -7.74E-02 | 4.97E-02 | 1.20E-01 | -6.18E-02 | 7.53E-02 | 4.12E-01 |

S5 Table. Summary statistics from regression analyses of unweighted and weighted gene scores on phenotypes.

|  | eGFR increasing allele | beta | se | p |
| --- | --- | --- | --- | --- |
| rs653178* | T | 0.016 | 0.224 | 9.42E-01 |
| rs6420094* | A | 0.054 | 0.326 | 8.67E-01 |
| rs3828890 | C | 0.107 | 0.379 | 7.77E-01 |
| rs17730281 | A | 0.11 | 0.338 | 7.44E-01 |
| rs1933182 | A | 0.134 | 0.243 | 5.83E-01 |
| rs7805747** | A | 0.323 | 0.282 | 2.51E-01 |
| rs881858** | A | 0.336 | 0.264 | 2.03E-01 |
| rs4744712* | C | 0.341 | 0.226 | 1.32E-01 |
| rs8101881 | T | 0.456 | 0.229 | 4.59E-02 |
| rs8068318 | C | 0.499 | 0.256 | 5.10E-02 |
| rs1260326* | T | 0.603 | 0.231 | 8.92E-03 |
| rs1731274 | G | 0.671 | 0.281 | 1.71E-02 |
| rs4014195 | G | 0.763 | 0.251 | 2.38E-03 |
| rs267734** | T | 0.856 | 0.276 | 1.94E-03 |
| rs17319721* | G | 0.961 | 0.225 | 2.04E-05 |
| rs12917707* | T | 1.291 | 0.286 | 6.43E-06 |
| rs10206899 | T | 1.837 | 0.76 | 1.57E-02 |

S6 Table. Internal weights estimated in UCLEB. Out of these 17 SNPs, 9 SNPs previously reported associations between eGFR-SNP in Olden et al., 2013 (*,**). Three of them are shown to have different effect direction from what we observed in UCLEB (**).

17 SNPs

UCLEB

(2,249 CHD cases and 10,896 controls)

yes

CARDIoGRAMplusC4D

(63,746 CHD cases and 130,681 controls)

Total samples that are used for each SNP to further investigate association with CHD

rs267734, rs4744712, rs653178, rs8101881

(65,995 cases and 141,577 controls)

no

rs1260326, rs6420094, rs7805747

(39,902 cases and 90,720 controls)

CARDIoGRAM GWAS & C4D GWAS

(37,653 cases and 79,824 controls)

no

rs1933182, rs10206899, rs17319721, rs881858, rs4014195, rs17730281, rs12917707, rs8068318

(24,482 cases and 75,658 controls)

CARDIoGRAM GWAS

(22,233 CHD cases and 64,762 controls)

rs3828890, rs1731274

(2,249 CHD cases and 10,896 controls)

S1 Figure. A flowchart representing total samples that are used for each SNP to further investigate association with CHD

**Supplementary Methods**

**Significance thresholds under multiple testing**

We observed substantial correlation between some traits measured by UCLEB and therefore suspected that the Benjamini-Hochberg method might be too conservative. To assess this we considered family-wise type-1 error and applied a permutation approach to identify 5% significance thresholds across multiple traits tested. These were then compared to Bonferroni corrections.

To simulate the null hypothesis of no association between eGFR and any of the 94 traits available in UCLEB, eGFR values were randomly shuffled among subjects within each cohort, to break any association between eGFR and traits. Then the permuted eGFR was linearly regressed across all 94 traits, and the minimum p-value across 94 null p-values was recorded. This was repeated 10000 times to obtain an empirical distribution of the minimum p-value under the null. The 5% quantile of this distribution is an estimate of the significance threshold required to obtain family-wise type-1 error of 5%. We repeated this procedure using CHD as the outcome and logistic regression for analysis.

To simulate the null hypothesis of no association between gene scores and any of the 94 traits available in UCLEB (test of possible pleiotropic effect of gene score), a similar approach was performed with trait values randomly shuffled among subjects.

The estimated 5% family-wise error thresholds are shown in Supplementary table VII, and are seen to be very similar to the Bonferroni threshold of 0.05/94=5.32×10^-4^. We concluded that the correlation could safely be ignored for family-wise error control, and assumed that the same could apply to false discovery rate control.

| Association between | 5% FWER |
| --- | --- |
| eGFR - 94 traits | 3.85x10^-4^ |
| CHD - 94 traits | 6.78x10^-4^ |
| unweighted gene score – 94 traits | 5.05x10^-4^ |
| internally weighted gene score – 94 traits | 4.34x10^-4^ |

S7 Table. Significance thresholds for 5% FWER estimated from permutation testing.
